# Comparison of AI-assisted cephalometric analysis and orthodontist-performed digital tracing analysis

**DOI:** 10.1186/s40510-024-00539-x

**Published:** 2024-10-21

**Authors:** Sabahattin Bor, Saadet Çınarsoy Ciğerim, Seda Kotan

**Affiliations:** 1 Faculty of Dentistry, Department of Orthodontics, İnönü University, Malatya, Turkey; 2 Faculty of Dentistry, Department of Orthodontics, Van Yüzüncü Yıl University, Van, Turkey

**Keywords:** AI-assisted cephalometric analysis, Angular and linear measurements, 2D lateral films, Diagnostic accuracy

## Abstract

**Background:**

The aim of this study was to compare and evaluate three AI-assisted cephalometric analysis platforms—CephX, WeDoCeph, and WebCeph—with the traditional digital tracing method using NemoCeph software.

**Material and method:**

A total of 1500 lateral cephalometric films that met the inclusion criteria were classified as Class I, Class II, and Class III. Subsequently, 40 patients were randomly selected from each class. These selected films were uploaded to 3 AI-assisted cephalometric analysis platforms and analyzed without any manual intervention. The same films were also analyzed by an orthodontist using the NemoCeph program.

**Results:**

The results revealed significant differences in key angular measurements (ANB, FMA, IMPA, and NLA) across Class I, II, and III patients when comparing the four cephalometric analysis methods (WebCeph, WeDoCeph, CephX, and NemoCeph). Notably, ANB (*p* < 0.05), FMA (*p* < 0.001), IMPA (*p* < 0.001), and NLA (*p* < 0.001) varied significantly. Linear measurements also differed, with significant differences in U1-NA (*p* = 0.002) and Co-A (*p* = 0.002) in certain classes. Repeated measurement analysis revealed variation in SNA (*p* = 0.011) and FMA (*p* = 0.030), particularly in the Class II NemoCeph group, suggesting method-dependent variability.

**Conclusion:**

AI-assisted cephalometric analysis platforms such as WebCeph, WeDoCeph, and CephX give rise to notable variation in accuracy and reliability compared to traditional manual digital tracing, specifically in terms of angular and linear measurements. These results emphasize the importance of meticulous selection and assessment of analysis methods in orthodontic diagnostics and treatment planning.

**Supplementary Information:**

The online version contains supplementary material available at 10.1186/s40510-024-00539-x.

## Introduction

Artificial intelligence (AI) refers to technological systems designed by humans, such as computers and robots, which imitate certain cognitive functions of humans, including reasoning, interpretation, and creative thinking [1]. Within the field of AI, machine learning stands out by focusing on the development of statistical models and algorithms that enable computers to learn and make predictions or decisions without being explicitly programmed [2].

If we were to define deep learning as a subset of machine learning, it uses artificial neural networks inspired by the human brain to automatically learn and extract hierarchical features from data, further enabling computers to make predictions or decisions independently [3]. One notable class of deep learning models is convolutional neural networks, which are specifically tailored for visual data analysis. These networks use convolutional operations to identify patterns and characteristics in images, employ shared parameters to simplify the model, incorporate pooling layers to reduce the size of feature maps, and utilize fully connected layers for classification or decision-making [1, 3].

This technology has been applied in several fields, including dentistry and orthodontics [4, 5]. In the field of orthodontics, AI has been used for dental monitoring, identification of hand–wrist or cervical vertebral maturation stages, segmentation of the jaw and teeth on cone-beam computed tomography (DICOM and STL files), treatment planning, and importantly, cephalometric analysis [4–27].

Cephalometric analysis plays an essential role in orthodontic and orthognathic surgeries, providing useful information for the diagnosis, planning, and evaluation of craniofacial growth and development. Numerous methods have been developed for cephalometric analysis, including manual acetate tracings, digital tracings on computers, and innovative approaches such as tracing on smartphone or iPad apps. The latest advancement in the field of cephalometric analysis is AI-assisted analysis using dedicated websites [22, 23, 26–31].

Computer-aided cephalometric analysis offers numerous benefits but is still dependent on human execution, which can be influenced by various individual factors such as eye fatigue, screen resolution, and the operator’s level of expertise [24, 32]. Unlike human operators, AI systems are not subject to physical or perceptual fatigue, which allows them to avoid many of the errors caused by human limitations [23]. They allow for the completion of cephalometric analyses in a short period of time [26, 33].

Accurate landmark identification is essential for true cephalometric analysis [27, 34]. Angular and linear measurements from the identified cephalometric landmarks are calculated automatically. Numerous studies have demonstrated that AI platforms can accurately identify these landmarks; however, there are few studies comparing the angular and linear measurements calculated by various AI platforms [18, 21, 30].

This study aimed to compare the accuracy and consistency of three AI-based cephalometric analysis platforms, WebCeph (version 1.5.0; Assemblecircle, Gyeonggi-do, Korea), WeDoCeph (Audax d.o.o., Ljubljana, Slovenia), and CephX (ORCA Dental AI Inc., Herzliya, Israel), with digital tracing performed by an experienced orthodontist using NemoCeph (Nemostudio Fall Edition 2020, Madrid, Spain).

## Materials and methods

This retrospective study analyzed 1,890 lateral cephalometric radiographs (Dentsply Sirona, Charlotte, NC, USA) from patients aged 12–18 years who underwent orthodontic treatment at the Department of Orthodontics, Yüzüncü Yıl University (YYU). The study protocol was approved by the ethical board of YYU. In the Department of Orthodontics at YYU, a total of 1,890 films were reviewed, of which 1,500 met the eligibility criteria.

### Inclusion criteria

- High-quality cephalograms that accurately demonstrated the cephalostat position without any artifacts that could obstruct the identification of anatomical sites.
- Individuals with Class I, Class II, and Class III malocclusions.
- Patients with cephalometric films obtained prior to orthodontic treatment.

### Exclusion criteria

- Cephalograms where the landmarks are not clearly defined.
- Cephalograms with significant double borders of the mandible.
- Individuals with craniofacial anomalies, asymmetries, or a history of craniofacial surgery.
- Individuals with significant dental abnormalities, diseases affecting cephalogram analysis, multiple missing teeth, or extensive crown-bridge restoration.

The eligible images were categorized according to ANB angle into three skeletal classes: Class I (0 < ANB < 4), Class II (ANB > 4), and Class III (ANB < 0). The distribution of images across these categories was as follows: 696 images in skeletal class I, 497 images in skeletal class II, and 307 images in skeletal class III. This cephalometric categorization was performed by an experienced orthodontist (S.C.Ç). The images in each group were numbered. Then, in each group, 40 images were randomly selected using Random Number Generator (https://www.calculatorsoup.com). The skeletal classes were renamed by a resident as Class I (Class Y), Class II (Class X), and Class III (Class Z), and the selected images were anonymized and sent to the orthodontist for cephalometric analysis.

120 images were uploaded by an experienced (11 year) orthodontist (S.B) to three AI platforms. On the WebCeph and WeDoCeph platforms, the calibration of the films was done manually. However, on the CephX platform, the images were calibrated by AI. There was no manual intervention in the identification of landmarks on the three AI platforms. After the cephalometric analyses were completed, the resulting values for linear and angular parameters were exported.

For the orthodontist-performed group, the cephalometric images were uploaded to the NemoCeph program by five orthodontic residency students who are in their final year of training. The tracings were conducted by an experienced (6 year) orthodontist (S.K) with no more than five images analyzed per day. Landmark identification was performed in collaboration with five residents, with the final decision made by the orthodontist. As the orthodontist made the final decision and the residents were still in training, the group was considered the orthodontist-performed group.

One month later, cephalometric images of 10 individuals from each skeletal class were uploaded again to the AI platforms under different names for subsequent analyses. The same orthodontist (S.K) repeated cephalometric analyses for these 30 individuals (10 per class) using the NemoCeph digital tracing program. Additionally, another experienced (12 year) orthodontist (Y.K) reanalyzed 30 randomly selected images from the set of 120 images and performed tracings in NemoCeph program.

The seven angular parameters (ANB, SNB, SNA, FMA, IMPA, NLA, and U1/SN) and seven linear parameters (U1/NA, L1/NB, Co-A, ANS-Me, Co-Gn, U-E line, and L-E line) (Table S1) were measured across four analysis groups. The groups were determined as follows: three AI-assisted groups—G1 (WebCeph), G2 (WeDoCeph), and G3 (CephX)—and one orthodontist group, labeled as G4 (NemoCeph).

### Statistical analysis

SPSS software (version 27.0; IBM Corp., Armonk, NY, USA) was used for statistical analysis. Descriptive statistic data was presented with mean, standard deviation, and maximum-minimum values. Statistical tests were conducted for each skeletal class. The Shapiro-Wilk test was used to examine the distribution of the data, while Levene’s test was used to assess homogeneity of variance. Since the data showed a normal distribution and equal variances, the one-way ANOVA, a parametric test, was used to evaluate comparisons between groups. Tukey’s post hoc test was subsequently applied to identify specific differences between groups. Additionally, the reliability of analysis methods was assessed using the Wilcoxon signed-rank test by comparing the initial and final analyses of 10 samples in each class. For the orthodontist-performed group, reliability was also checked using Intraclass correlation coefficient for both intra-rater and inter-rater reliability. The Intraclass Correlation Coefficient (ICC) value is considered good when it is between 0.75 and 0.90, and excellent when it is above 0.90 [23].

## Results

The intra-rater assessment of the orthodontist-performed group revealed that in Class I, the FMA; in Class II, the NLA; and in Class III, the ANB and FMA had ICC values below 0.75, indicating less reliable results. Other parameters generally demonstrated good to excellent agreement. For linear parameters, Co-A in Class II, and Co-A and Co-Gn in Class III showed poor agreement, while most other parameters had excellent agreement. In the inter-rater ICC values, the FMA and NLA angles in Class II, and the U-E line in Class III also showed agreement below 0.75, with other parameters exhibiting good to excellent agreement.

In class I, there were significant differences in angular measurements for ANB (*p* < 0.05), FMA (*p* < 0.001), IMPA (*p* < 0.001), and NLA (*p* < 0.001) among cephalometric analysis groups. In the WebCeph group, the mean ANB was 3.83˚ and the highest IMPA was 92.97˚. Conversely, in the NemoCeph group, the highest mean FMA was 28.8˚. Additionally, the peak NLA was 111.13˚ in the CephX group. There were no significant differences among the groups in any other angular measurements (*p* > 0.05).

In class II, significant differences were observed in the angular measurements for ANB (*p* < 0.05), FMA (*p* < 0.05), IMPA (*p* < 0.05), and NLA (*p* < 0.001) among the cephalometric analysis groups. The WebCeph group exhibited the highest ANB (6.63˚) and IMPA (99.01˚). In the NemoCeph group, the highest FMA was 28.71˚. Furthermore, the CephX group exhibited the highest NLA (109.61˚).

In class III, significant differences were observed among cephalometric analysis methods in terms of angular measurements of ANB (*p* < 0.05), FMA (*p* < 0.05), IMPA (*p* < 0.05), and NLA (*p* < 0.001). The NemoCeph group exhibited the lowest ANB (− 1.95˚) and highest FMA (28.88˚) and IMPA (85.65˚). Additionally, the CephX group demonstrated the highest NLA (106.25˚) (Table 1).

**Table 1:** Comparison of angular measurements by cephalometric analysis methods

	Class I					Class II					Class III				
Measurements (Degree)	G1 M ± SD (Min-Max)	G2 M ± SD (Min-Max)	G3 M ± SD (Min-Max)	G4 M ± SD (Min-Max)	*p*-value	G1 M ± SD (Min-Max)	G2 M ± SD (Min-Max)	G3 M ± SD (Min-Max)	G4 M ± SD (Min-Max)	*p*-value	G1 M ± SD (Min-Max)	G2 M ± SD (Min-Max)	G3 M ± SD (Min-Max)	G4 M ± SD (Min-Max)	*p*-value
SNA	81.74 ± 3.23 (76–87)	80.48 ± 3.46 (74–87)	80.95 ± 3.41 (74–87)	81.33 ± 3.47 (73–87)	0.395	81.77 ± 3.29 (74–90)	80.75 ± 3.23 (74–89)	80.95 ± 3.1 (90–81)	80.9 ± 3.3 (73–90)	0.490	80.92 ± 3.14 (74–87)	80.22 ± 3.75 (72–86)	80.35 ± 3.57 (73–88)	80.58 ± 3.95 (72–89)	0.834
SNB	77.89 ± 3.09 (71–84)	77.92 ± 3.37 (71–84)	78.11 ± 3.31 (71–85)	78.8 ± 3.29 (71–84)	0.569	75.14 ± 3.18 (68–83)	75.21 ± 3.1 (67–84)	75.31 ± 3.15 (85–75)	75.7 ± 3.28 (66–85)	0.863	81.4 ± 3.38 (74–88)	81.9 ± 3.87 (75–90)	82.03 ± 3.94 (76–91)	82.55 ± 4.19 (75–93)	0.617
ANB	3.83 ± 1.69 (0–7)	2.57 ± 1.78 (− 2–5)	2.83 ± 1.69 (0–6)	2.53 ± 1.62 (− 2–5)	0.002*	6.63 ± 1.67 (4–11)	5.54 ± 1.75 (2–9)	5.64 ± 1.74 (9–6)	5.23 ± 2.15 (1–9)	0.006*	-0.48 ± 1.75 (-6–3)	-1.69 ± 2.11 (-8–4)	-1.66 ± 2.02 (-8–2)	-1.95 ± 2.09 (-7–3)	0.006*
FMA	23.91 ± 4.76 (14–34)	25.85 ± 4.97 (17–36)	25.14 ± 4.25 (18–34)	28.8 ± 5.47 (18–45)	0.000*	24.54 ± 5.84 (10–35)	26.01 ± 6.68 (6–36)	25.55 ± 5.2 (35–25)	28.71 ± 5.84 (17–40)	0.014*	25.63 ± 5.01 (17–37)	27.02 ± 7.08 (-4–39)	25.63 ± 4.61 (17–36)	28.88 ± 6.26 (10–42)	0.042*
IMPA	92.97 ± 5.58 (82–106)	89.75 ± 6.05 (79–103)	86.05 ± 5.56 (72–100)	92.13 ± 6.46 (76–104)	0.000*	99.01 ± 6.34 (86–112)	95.87 ± 6.8 (79–110)	92.62 ± 6.47 (107–92)	97.02 ± 7.61 (80–115)	0.001*	85.09 ± 6.36 (66–103)	81.79 ± 6.75 (64–101)	80.3 ± 6.54 (59–97)	85.65 ± 7.69 (67–106)	0.001*
U1/NA	19.18 ± 4.82 (9–28)	20.15 ± 5.27 (9–31)	21.66 ± 4.06 (13–32)	20.78 ± 4.83 (12–32)	0.129	21.46 ± 6.59 (9–32)	23.4 ± 7.34 (10–37)	24.87 ± 6.42 (37–27)	24.58 ± 6.76 (12–38)	0.105	24.17 ± 4.35 (16–34)	24.65 ± 5.6 (10–34)	26.74 ± 4.4 (18–35)	25.78 ± 6.41 (10–38)	0.128
L1/NB	24.02 ± 3.92 (14–33)	23.74 ± 5.39 (8–35)	21.72 ± 4.93 (5–34)	23.35 ± 5.31 (13–32)	0.160	28.12 ± 4.96 (18–38)	27.87 ± 5.99 (14–39)	26.28 ± 5.96 (37–27)	26.42 ± 6.39 (13–39)	0.364	20.2 ± 5.79 (6–32)	20.11 ± 6.37 (6–35)	19.08 ± 6.31 (1–33)	21.2 ± 7.91 (5–37)	0.566
NLA	99.03 ± 13.36 (78–132)	107.35 ± 11.89 (87–137)	111.13 ± 11.32 (91–136)	104.68 ± 14.61 (78–135)	0.000*	99.37 ± 9.77 (69–114)	106.7 ± 9.29 (85–121)	109.61 ± 10.26 (135–112)	105.13 ± 9.91 (76–125)	0.000*	94.98 ± 10.15 (66–114)	101.53 ± 10.68 (75–131)	106.25 ± 8.73 (90–130)	98.75 ± 12.63 (63–131)	0.000*
U1/SN	100.91 ± 5.78 (91–113)	100.64 ± 6.16 (90–113)	102.61 ± 5.54 (92–117)	102.28 ± 5.3 (92–113)	0.319	103.23 ± 6.87 (87–113)	104.14 ± 7.71 (85–117)	105.82 ± 6.58 (117–107)	105.57 ± 7.15 (89–119)	0.313	105.08 ± 5.86 (93–116)	104.87 ± 7.08 (83–117)	107.1 ± 6.13 (95–116)	106.38 ± 8.01 (82–119)	0.409

*p < 0.05 indicates a significant difference, whereas *p* > 0.05 indicates no significant difference by one-way ANOVA. **M**: Mean, **SD**: Standard Deviation, **Min-Max**: Minimum-Maximum. **mm =** milimeter. G1, G2, and G3 are AI-assisted platforms, while G4 is a traditional digital program. **G1**: WebCeph, **G2**: WeDoCeph, **G3**: CephX, **G4**: NemoCeph

In class I, a comparison of cephalometric analysis methods revealed statistically significant differences in certain linear measurements, including U1-NA (*p* < 0.001), Co-A (*p* = 0.001), and Co-Gn (*p* = 0.001). In particular, the NemoCeph group exhibited the highest U1-NA (3.67 mm) and Co-Gn (105.48 mm) values, whereas the WeDoCeph group demonstrated the highest Co-A (80.14 mm) value.

In class II, significant differences were observed in specific linear parameters among the cephalometric analysis methods, including U1-NA (*p* = 0.014), Co-A (*p* < 0.001), and Co-Gn (*p* = 0.001). The highest Co-A value (81.45 mm) was found in the WeDoCeph group, while the highest U1-NA (4.91) and Co-Gn (102.05 mm) values were recorded in the NemoCeph group.

In Class III, significant differences were observed in specific linear parameters among the cephalometric analysis methods, including U1-NA (*p* = 0.002) and Co-A (*p* = 0.002). The highest Co-A value (76.15 mm) was recorded in the WeDoCeph group, while the highest U1-NA value (4.97 mm) was found in the CephX group (Table 2).

**Table 2:** Comparison of Linear measurements by cephalometric analysis methods

	Class I					Class II					Class III				
Measurements (mm)	G1 M ± SD (Min-Max)	G2 M ± SD (Min-Max)	G3 M ± SD (Min-Max)	G4 M ± SD (Min-Max)	*p*-value	G1 M ± SD (Min-Max)	G2 M ± SD (Min-Max)	G3 M ± SD (Min-Max)	G4 M ± SD (Min-Max)	*p*-value	G1 M ± SD (Min-Max)	G2 M ± SD (Min-Max)	G3 M ± SD (Min-Max)	G4 M ± SD (Min-Max)	*p*-value
U1-NA	3.32 ± 2.05 (0–7)	4.82 ± 2.49 (0–9)	4.64 ± 2.43 (9–5)	4.91 ± 2.81 (− 1–12)	0.015*	3.32 ± 2.05 (0–7)	4.82 ± 2.49 (0–9)	4.64 ± 2.43 (9–5)	4.91 ± 2.81 (− 1–12)	0.014*	3.22 ± 1.73 (0–6)	4.23 ± 2.51 (–3–8)	4.97 ± 1.57 (1–8)	4.65 ± 2.39 (1–11)	0.002*
L1-NB	5.62 ± 2.11 (2–10)	5.65 ± 2.44 (2–11)	4.92 ± 2.3 (10–5)	4.94 ± 2.38 (0–9)	0.085	5.62 ± 2.11 (2–10)	5.65 ± 2.44 (2–11)	4.92 ± 2.3 (10–5)	4.94 ± 2.38 (0–9)	0.303	3.31 ± 1.9 (0–7)	3.2 ± 2.06 (− 1–7)	2.5 ± 2.27 (− 2–7)	3.06 ± 2.04 (− 1–7)	0.304
Co-A	78.59 ± 3.15 (72–85)	81.45 ± 3.38 (75–89)	77.85 ± 3 (83–78)	80.79 ± 4.41 (71–89)	0.000*	78.59 ± 3.15 (72–85)	81.45 ± 3.38 (75–89)	77.85 ± 3 (83–78)	80.79 ± 4.41 (71–89)	0.000*	74.03 ± 4.02 (64–82)	76.15 ± 4.38 (68–86)	72.46 ± 4.08 (66–82)	74.97 ± 5.05 (65–87)	0.002*
ANS-Me	60.22 ± 5.81 (51–73)	60.84 ± 6.04 (51–76)	60.37 ± 5.57 (71–59)	60.38 ± 6.28 (48–76)	0.882	60.22 ± 5.81 (51–73)	60.84 ± 6.04 (51–76)	60.37 ± 5.57 (71–59)	60.38 ± 6.28 (48–76)	0.970	58.43 ± 4.84 (50–70)	59.22 ± 4.83 (50–72)	58.83 ± 4.91 (50–70)	59.33 ± 5.71 (46–73)	0.854
Co-Gn	99.21 ± 5.15 (90–111)	101.43 ± 5.79 (91–114)	97.42 ± 5.08 (112–98)	102.05 ± 6.5 (88–117)	0.001*	99.21 ± 5.15 (90–111)	101.43 ± 5.79 (91–114)	97.42 ± 5.08 (112–98)	102.05 ± 6.5 (88–117)	0.001*	103.59 ± 7.62 (86–122)	105.14 ± 7.88 (89–124)	101.59 ± 7.7 (88–123)	105.05 ± 8.31 (90–126)	0.154
U-E line	0.11 ± 2.1 (− 5–4)	−0.53 ± 2.04 (− 6–3)	0.02 ± 2.23 (4–0)	−0.5 ± 2.02 (− 6–3)	0.594	0.11 ± 2.1 (− 5–4)	−0.53 ± 2.04 (− 6–3)	0.02 ± 2.23 (4–0)	−0.5 ± 2.02 (− 6–3)	0.379	−4.07 ± 2.51 (− 12––1)	−4.76 ± 2.39 (− 11–2)	−4.4 ± 2.59 (− 12–0)	−4.59 ± 3.12 (− 17–1)	0.686
L-E line	0.46 ± 2.35 (− 6–6)	0.67 ± 2.63 (− 6–6)	1.23 ± 2.54 (7–1)	0.64 ± 2.5 (− 6–6)	0.838	0.46 ± 2.35 (− 6–6)	0.67 ± 2.63 (− 6–6)	1.23 ± 2.54 (7–1)	0.64 ± 2.5 (− 6–6)	0.5	−0.95 ± 2.9 (− 7–5)	−0.67 ± 3.12 (− 7–4)	−0.93 ± 3.11 (− 7–5)	−0.67 ± 2.47 (− 7–4)	0.952

*p < 0.05 indicates a significant difference, whereas *p* > 0.05 indicates no significant difference by one-way ANOVA. **M**: Mean, **SD**: Standard Deviation, **Min-Max**: Minimum-Maximum. **mm** = milimeter. G1, G2, and G3 are AI-assisted platforms, while G4 is a traditional digital program. **G1**: WebCeph, **G2**: WeDoCeph, **G3**: CephX, **G4**: NemoCeph

**Table 3:** Pairwise comparison of angular measurements between groups in different skeletal classes

Class	Measurements	G1-G2	G1-G3	G1-G4	G2-G3	G2-G4	G3-G4
Class I	ANB ˚	0.006*	0.045*	0.004*	0.897	0.999	0.849
	FMA ˚	0.290	0.676	0.000*	0.915	0.038*	0.005*
	IMPA ˚	0.075	0.000*	0.919	0.030*	0.279	0.000*
	NLA ˚	0.022*	0.000*	0.207	0.557	0.788	0.116
Class II	ANB ˚	0.043*	0.080	0.005*	0.995	0.875	0.749
	FMA ˚	0.682	0.871	0.010*	0.985	0.177	0.083
	IMPA ˚	0.171	0.000*	0.560	0.148	0.876	0.023*
	NLA ˚	0.006*	0.000*	0.046*	0.546	0.892	0.177
Class III	ANB ˚	0.039*	0.044*	0.007*	0.999	0.934	0.919
	FMA ˚	0.709	0.999	0.065	0.711	0.485	0.065
	IMPA ˚	0.140	0.011*	0.984	0.767	0.061	0.004*
	NLA ˚	0.033*	0.000*	0.390	0.198	0.649	0.010*

*p < 0.05 indicates a statistically significant difference, whereas *p* > 0.05 indicates no statistically significant difference by Tukey’s test. G1, G2, and G3 are AI-assisted platforms, while G4 is a traditional digital program. **G1**: WebCeph, **G2**: WeDoCeph, **G3**: CephX, **G4**: NemoCeph

**Table 4:** Pairwise comparison of Linear measurements between groups in different skeletal classes

Class	Measurements	G1-G2	G1-G3	G1-G4	G2-G3	G2-G4	G3-G4
Class I	U1-NA mm	0.062	0.037*	0.032*	0.997	0.994	0.999
	Co-A mm	0.011*	0.587	0.065	0.000*	0.915	0.001*
	Co-Gn mm	0.371	0.385	0.142	0.009*	0.951	0.001*
Class II	U1-NA mm	0.036*	0.080	0.022*	0.989	0.998	0.962
	Co-A mm	0.002*	0.787	0.030*	0.000*	0.842	0.002*
	Co-Gn mm	0.300	0.492	0.116	0.010*	0.961	0.002*
Class III	U1-NA mm	0.140	0.001*	0.013*	0.383	0.797	0.903
	Co-A mm	0.142	0.387	0.776	0.001*	0.630	0.057

*p < 0.05 indicates a statistically significant difference, whereas *p* > 0.05 indicates no significant difference by Tukey’s test. G1, G2, and G3 are AI-assisted platforms, while G4 is a traditional digital program. **G1**: WebCeph, **G2**: WeDoCeph, **G3**: CephX, **G4**: NemoCeph

Post hoc Tukey’s test was used for pairwise comparisons of cephalometric analysis groups that showed statistically significant differences in one-way ANOVA (Tables 3 and 4).

### Repeated measurements

We compared the initial and final angular measurements of 10 patients of each skeletal class using the Wilcoxon signed-rank test. There were no significant differences among the AI-based groups, whereas the NemoCeph group, which involved the use of a digital manual approach, demonstrated significant differences between the initial and final SNA and FMA.

**Table 5:** Comparison of repeated linear measurements according to cephalometric analysis methods in different skeletal classes

	Class I				Class II				Class III			
	**G1**	**G2**	**G3**	**G4**	**G1**	**G2**	**G3**	**G4**	**G1**	**G2**	**G3**	**G4**
U1-NA	0.107	0.18	0.999	0.021*	0.153	0.999	0.999	0.043*	0.019*	0.083	0.999	0.878
L1-NB	0.098	0.18	0.999	0.055	0.134	0.317	0.999	0.999	0.024*	0.317	0.999	0.682
Co-A	0.284	0.256	0.999	0.018*	0.139	0.056	0.999	0.074	0.012*	0.03*	0.999	0.047*
ANS-Me	0.126	0.084	0.999	0.916	0.037	0.091	0.999	0.333	0.012*	0.034*	0.999	0.441
Co-Gn	0.203	0.212	0.999	0.018*	0.114	0.067	0.999	0.021*	0.012*	0.021*	0.999	0.011*
U-E Line	0.056	0.317	0.999	0.916	0.832	0.999	0.999	0.191	0.062	0.157	0.999	0.406
L-E Line	0.033*	0.317	0.999	0.028*	0.683	0.157	0.999	0.721	0.169	0.838	0.047*	0.221

*p < 0.05 indicates a statistically significant difference, whereas *p* > 0.05 indicates no significant difference by Wilcoxon signed-rank test. G1, G2, and G3 are AI-assisted platforms, while G4 is a traditional digital program. **G1**: WebCeph, **G2**: WeDoCeph, **G3**: CephX, **G4**: NemoCeph

We analyzed the initial and final measurements obtained using WebCeph (G1), WeDoCeph (G2), CephX (G3), and NemoCeph (G4). There were significant differences in linear measurements among the three groups. WebCeph demonstrated no significant differences in measurements for class I, whereas significant differences were observed in ANS-Me for class II and in all measurements except U-E line and L-E line for class III. Similar to WebCeph, WeDoCeph did not demonstrate any significant differences in measurements for class I, whereas significant differences were observed in L1-NB for class II and in Co-A, ANS-Me, and Co-Gn for class III.

CephX provided the best consistency in measurements, with no significant differences in any class. Conversely, NemoCeph provided variable results, with significant differences in Co-A, Co-Gn, and L-E line for class I; Co-A and Co-Gn for class III; and U1-NA and Co-Gn for class II (Table 5).

## Discussion

In orthodontics, cephalometric analysis provides crucial information for treatment planning, progress monitoring, and outcome evaluation. Accurate analyses are essential for developing appropriate treatment plannings and minimizing the risk of erroneous interventions. To ensure patient safety, the validation of AI, with reference to traditional methodologies, is necessary for the successful integration of AI into orthodontic treatment.

In our study, we evaluated both angular and linear parameters commonly used in orthodontic treatment planning, building on previous assessments to enable comparison with other studies. By conducting a comprehensive analysis of multiple AI platforms, we aimed to minimize bias and assess the potential utility of AI in cephalometric analysis.

Previous studies have investigated whether AI platforms can accurately detect cephalometric landmarks [21, 25, 34]. It has suggested that a deviation in the cephalometric point < 2 mm is acceptable [27, 35]. Therefore, many studies have found AI platforms to be accurate for landmark detection [18, 20, 21]. However, deviations < 2 mm in three-point angular measurements can lead to significant differences.

Kunz et al. [30] conducted a study comparing the performance of four AI platforms with that of 12 human professionals, who served as the gold standard. Among the platforms, DentaliQ.ortho’s AI demonstrated the highest agreement with the human experts. While WebCeph also showed strong overall agreement, it exhibited lower precision in its measurements. In contrast, CephX and AudaxCeph showed significant deviations in their cephalometric analyses, particularly in key parameters compared to the human experts. This was the first study to compare multiple AI platforms.

In our study, we used the same three AI-assisted platforms, with the exception of DentaliQ.ortho.We expanded our analysis by using a larger dataset and comparing the AI platforms in different skeletal classes. Contrary to the previous study’s findings, our results showed that WeDoCeph (AudaxCeph) provided outcomes more consistent with the orthodontist group for angular measurements in all skeletal classes. In linear measurements, the WeDoCeph group also yielded results more consistent with the orthodontist group, while both the WebCeph and CephX groups showed significant differences in a total of four parameters across all skeletal classes compared to the orthodontist group.

When comparing the AI platforms to each other, we observed significant differences in angular measurements, particularly between WebCeph and CephX. Although WeDoCeph and CephX showed significant differences in the IMPA angle across all skeletal classes, they still produced compatible results overall. However, this compatibility was not observed in linear measurements. We attribute this difference to the calibration methods used by the platforms: CephX uses artificial intelligence for calibration, whereas WeDoCeph employs manual calibration, which may explain the significant differences between these two platforms in linear measurements.

Our findings underscore that calibration procedures can significantly impact the accuracy of linear measurements. Automated calibration, as employed by CephX, can reduce discrepancies and ensure more consistent results, especially in repeated measurements.

Despite the differences among AI platforms, the classification of the sagittal direction was generally consistent. This finding is similar to the results of Yu et al. [36] However, variations in orthodontic classifications, particularly in class III, emphasize the need for customized AI approaches in complex cases to improve accuracy and reliability.

For class I–III patients, significant differences were observed in linear measurements, such as U1-NA, Co-A, and Co-Gn, among WebCeph, WeDoCeph, CephX, and NemoCeph. The highest Co-Gn value was observed in WebCeph and NemoCeph groups for class I and in WebCeph for class II. Furthermore, in class III, the highest U1-NA value was observed in the CephX group. These results highlight the impact of platform-specific characteristics, which can influence orthodontic assessments and treatment planning.

The differences in Co-A and Co-Gn values among AI platforms demonstrate variability in the selection of the Co point, as reported by a previous study [37]. Furthermore, a significant difference was observed in the nasolabial angle, a soft tissue parameter, measured by various methods in all skeletal classes.

Minimal variations were observed between the initial and final measurements in the AI-assisted analysis groups. A similar outcome has been reported in another study [21]. However, in the orthodontist-performed group, both intra-rater and inter-rater assessments, particularly the intra-rater evaluations, showed discrepancies in certain parameters. This emphasizes that AI platforms provide more consistent analyses compared to the orthodontist-performed group.

While AI platforms offer greater consistency, the significant differences in angular and linear measurements between the AI platforms are likely due to data and algorithmic biases. Data bias can occur when the training datasets used by the AI systems are not fully representative of all possible scenarios or contain inherent biases, leading to skewed results. Algorithmic bias arises from how AI algorithms are designed and implemented, potentially overlooking certain variations in the data.

In our study, using AI platforms for landmark identification without manual editing is recognized as a limitation due to the potential inclusion of incorrectly identified landmarks. For future research, we recommend comparing scenarios with and without manual intervention by orthodontists to assess the impact on accuracy.

Additionally, although the landmarks were identified by an experienced orthodontist in collaboration with five residents, we acknowledge the limitation of involving only one orthodontist. To enhance the robustness of future studies, we suggest including a larger group of orthodontists to provide broader expertise and validation.

## Conclusion

There were significant differences in angular (ANB, FMA, IMPA, and NLA) and linear (U1-NA and Co-A) measurements obtained using AI-assisted cephalometric analysis (WebCeph, WeDoCeph, and CephX) and traditional manual digital tracing (NemoCeph). Repeated measurements demonstrated significant differences, particularly in the class II NemoCeph group, which was influenced by the analysis method. Although the use of AI-assisted platforms saves time and prevents human errors, the analysis requires human supervision by an orthodontist.

## Electronic supplementary material

Below is the link to the electronic supplementary material.

Supplementary Material 1

## Data Availability

The datasets used and/or analyzed during the current study are available from the corresponding author on reasonable request.
