# Supplementary material for: Comparison of AI-assisted cephalometric analysis and orthodontist-performed digital tracing analysis
Source: Prog Orthod. 2024 Oct 21;25:41. doi: 10.1186/s40510-024-00539-x (PMC11491421; doi:10.1186/s40510-024-00539-x)
Supplement: Supplementary file 1 — Supplementary Material 1 [file 40510_2024_539_MOESM1_ESM.docx]

Table S1: Angular and Linear Measurements

| **Angular measurement** | **Definition** |
| --- | --- |
| ANB | Angle formed between points A, N, and B; indicates the anteroposterior relationship between the maxilla and mandible |
| SNB | Angle formed between sella (S), nasion (N), and point B; indicates the anteroposterior position of the mandible |
| SNA | Angle formed between sella (S), nasion (N), and point A; indicates the anteroposterior position of the maxilla |
| Frankfort mandibular plane angle (FMA) | Angle formed between the Frankfort horizontal plane and the mandibular plane; indicates the vertical facial growth pattern |
| Incisor mandibular plane angle (IMPA) | Angle formed between the lower incisor axis and mandibular plane; indicates the lower incisor position |
| Nasolabial angle (NLA) | Angle formed between lines tangential to the base of the nose and upper lip; indicates the soft tissue profile |
| U1/NA | Angle formed between the upper incisor axis and NA line (nasion to A); indicates the upper incisor position |
| L1/NB | Angle formed between the lower incisor axis and NB line (nasion to B); indicates the lower incisor position |
| U1/SN | Angle formed between the upper incisor axis and SN (sella-nasion) plane; indicates the upper incisor position |
| **Linear measurement** | **Definition** |
| Condylion to point A (Co-A) | Linear measurement from the most posterior point on the mandibular condyle to point A; indicates the effective length of the maxilla |
| Anterior nasal spine to menton (ANS-Me) | Linear measurement from the anterior nasal spine to the most inferior point on the mandibular symphysis; indicates the height of the anterior lower face |
| Condylion to gnathion (Co-Gn) | Linear measurement from the most posterior point on the mandibular condyle to the most anterior-inferior point on the mandibular symphysis; indicates the effective length of the mandible |
| U1-NA | Horizontal distance between the tip of the upper central incisor (U1) and the perpendicular NA line, which extends from the nasal point to point A |
| L1-NB | Distance between the lower central incisor (L1) and the NB line, which connects the nasion and point B; indicates the position of the lower incisors |
| E-line | An aesthetic line connecting the pronasale and soft tissue pogonion |
| Upper lip to E-line (U-E line) | Linear distance between the most anterior point of  the upper lip and the E-line |
| Lower lip to E-line (L-E line) | Linear distance between the most anterior point of  the lower lip and the E-line |
